# WARE: Wet AMD Risk-Evaluation Tool as a Clinical Decision-Support System Integrating Genetic and Non-Genetic Factors

**DOI:** 10.3390/jpm12071034

**Published:** 2022-06-24

**Authors:** Carlo Fabrizio, Andrea Termine, Valerio Caputo, Domenica Megalizzi, Stefania Zampatti, Benedetto Falsini, Andrea Cusumano, Chiara Maria Eandi, Federico Ricci, Emiliano Giardina, Claudia Strafella, Raffaella Cascella

**Affiliations:** 1 Data Science Unit, IRCCS Santa Lucia Foundation c/o CERC, 00143 Rome, Italy; c.fabrizio@hsantalucia.it (C.F.); a.termine@hsantalucia.it (A.T.); 2 Genomic Medicine Laboratory UILDM, IRCCS Santa Lucia Foundation, 00179 Rome, Italy; v.caputo91@gmail.com (V.C.); domenica.megalizzi.96@gmail.com (D.M.); stefania.zampatti@gmail.com (S.Z.); claudia.strafella@gmail.com (C.S.); 3 Ophthalmology Unit, Fondazione Policlinico Universitario A. Gemelli IRCCS, 00168 Rome, Italy; benedetto.falsini@unicatt.it; 4 Department of Experimental Medicine, University of Rome Tor Vergata, Via Montpellier, 00133 Rome, Italy; cusumano@cusumano.com; 5 Department of Surgical Science, University of Torino, 10124 Torino, Italy; chiara.eandi@unito.it; 6 UOSD Retinal Diseases, PTV Foundation, University of Rome Tor Vergata, 00133 Rome, Italy; rccfrc00@gmail.com; 7 Department of Biomedicine and Prevention, University of Rome Tor Vergata, 00133 Rome, Italy; raffaella.cascella@uniroma2.it; 8 Department of Biomedical Sciences, Catholic University Our Lady of Good Counsel, 1000 Tirana, Albania

**Keywords:** AMD, aging, tool, risk evaluation, genetic and non-genetic factors, personalized approach

## Abstract

Given the multifactorial features characterizing age-related macular degeneration (AMD), the availability of a tool able to provide the individual risk profile is extremely helpful for personalizing the follow-up and treatment protocols of patients. To this purpose, we developed an open-source computational tool named WARE (Wet AMD Risk Evaluation), able to assess the individual risk profile for wet AMD based on genetic and non-genetic factors. In particular, the tool uses genetic risk measures normalized for their relative frequencies in the general population and disease prevalence. WARE is characterized by a user-friendly web page interface that is intended to assist clinicians in reporting risk assessment upon patient evaluation. When using the tool, plots of population risk distribution highlight a “low-risk zone” and a “high-risk zone” into which subjects can fall depending on their risk-assessment result. WARE represents a reliable population-specific computational system for wet AMD risk evaluation that can be exploited to promote preventive actions and personalized medicine approach for affected patients or at-risk individuals. This tool can be suitable to compute the disease risk adjusted to different populations considering their specific genetic factors and related frequencies, non-genetic factors, and the disease prevalence.

## 1. Introduction

The role of biomedical research and innovation in the discovery and development of new protocols is essential to quickly and effectively respond to the different needs of patients and relatives. On this subject, the realization and implementation of molecular assays combined with computational analysis methods stand out as crucial to fulfil the demand for effective preventive, diagnostic, prognostic, and treatment strategies of disease conditions [1,2]. In this perspective, the design of new strategies and personalized medicine represent a powerful tool for preventing the establishment of disease conditions and promoting the preservation of health and quality of life. Among the main disease causes, aging represents the key factor responsible for the gradual decline and subsequent onset of age-related pathologies including cancer, Alzheimer’s disease, Parkinson’s disease, rheumatoid arthritis, and maculopathies. Among the different forms of maculopathy, age-related macular degeneration (AMD) is the principal leading cause of visual impairment among people over 60 years old and living in developed countries. AMD affects approximately 170 million individuals worldwide and is predictable to double by 2050 [3]. In the United States, AMD prevalence is similar to that of invasive cancers, resulting in a direct healthcare cost of USD 4.6 billion annually [4]. In Italy, this disease affects about 1 million people [5].

AMD is caused by retinal degeneration, which impairs central vision, inducing visual acuity loss, images distortion, central scotoma, and altered perception of colors [3]. This condition strongly compromises patients’ daily activities and quality of life (driving, face recognition, reading, and working with fine detail). According to the AMD international classification, early, intermediate, and late stages of disease can be distinguished. In early and intermediate stage AMD, fundus examination reveals the presence of non-exudative lesions (drusen), which occur with significant pigment changes in the posterior pole. On the other hand, the late stage of AMD is characterized by either the formation of central retinal atrophy (geographic atrophy, GA) or the presence of an exudative lesion (choroidal neovascularization, CNV). In particular, the exudative form (here reported as wet AMD) is broadly acknowledged as a major cause of severe vision loss, usually occurring over weeks to months. In fact, wet AMD is typically characterized by angiogenesis and increased vascular permeability, both of which result from VEGF upregulation. Indeed, VEGF drives growth of neovessels sprouting from the choriocapillaris and spreading into the sub-retinal pigment epithelium or subretinal space. Given its prominent role in AMD etiopathogenesis, VEGF has been targeted by several drugs (namely, anti-VEGF inhibitors), which dramatically improved the prognosis of AMD although many patients do not respond adequately or experience a loss of efficacy after repeated administration [6,7].

It is well-known that a complex interplay among genetic and non-genetic factors (smoking habits, lifestyle, aging, diet, family history) contributes to the onset of AMD [8,9,10,11]. The impact of these risk factors has been extensively explored among worldwide populations, highlighting prominent differences [12]. Indeed, a number of susceptibility variants described in European, American, and Asian populations were not significantly associated in the Italian cohort. This scenario suggests the existence of a population-specific effect of such variants on the disease onset and progression. On this subject, a large-scale genotyping analysis including 1976 Italian subjects identified eight risk variants (rs1061170, T/C; rs10490924, G/T; rs2227306, C/T; rs5749482, C/G; rs8135665, C/T; rs8017304, A/G; rs943080, C/T; rs13081855, G/T) located within different genes (*CFH*, *ARMS2*, *IL-8*, *TIMP3*, *SLC16A8*, *RAD51B*, *VEGFA,* and *COL8A1*) known to be involved in the etiopathogenesis of wet AMD [5]. In fact, these data suggest that *CFH* (1q31), *ARMS2* (10q26.16), *IL-8* (4q12-q13), *TIMP3* (22q12.3), *SLC16A8* (22q13.1), *RAD51B* (14q23), *VEGFA* (6p21.1), and *COL8A1* (3q12.1) may contribute to the onset of wet AMD at different levels. The main related pathways include the angiogenesis, the alteration of extra cellular matric (ECM) remodeling mechanisms and of Bruch membrane (BrM) integrity and permeability, the modification of retinal pigment epithelium (RPE) and photoreceptor cell activities, and over-activation of inflammatory and immune responses. Furthermore, our previous works provided interesting insights into the interactions among AMD-associated genes, which may thereby trigger alterations of BrM structure and permeability, the induction of angiogenesis, and the damage caused by non-genetic factors [12]. Concerning the latter ones, a number of triggering factors have been described to date. First, aging is responsible for the loss of approximately 30% of rod photoreceptors and has been directly correlated with AMD prevalence. Aging tissues are known to be affected by a substantial increase in reactive oxygen species (ROS) levels and a reduction in antioxidants, which altogether make the retina more susceptible to oxidative damage. It has been estimated that the risk of AMD is influenced by the age, ranging from 1 in 56–69-year-old people to 4.42 in 70–79-year-old people up to 18.8–32.3 in 80–86-year-old people [12]. Second, smoking is known to significantly increase the risk for AMD as a result of the RPE damage and the reduction of choroidal blood flow caused by the release of oxidative compounds present in cigarettes [12]. Smoking consumes vitamins and micronutrients that work as essential antioxidant elements. Moreover, smoking has been supposed to induce ischemic, hypoxia, and micro-infarction events, which contribute to increase the susceptibility to AMD. Smoking has been associated with both exudative and non-exudative AMD although the correlation with the wet form proved to be stronger. Third, dietary habits, especially the consumption of food with antioxidant properties, are usually associated with slower disease progression towards more severe forms. In fact, several studies have shown that oral supplementation of antioxidants nutrients is able to prevent the progression of disease to more advanced stages. Among the different antioxidant compounds, the most investigated are carotenoids (lutein and zeaxanthin, β-carotene); vitamins (vitamin A, E, C, D, B); mineral supplements (zinc, copper, selenium); and omega-3 fatty acids [12]. In particular, antioxidant nutrients have been thought to act as “scavengers” by degrading the oxidized compounds that accumulate in the macula and are responsible of the RPE disruption and macular degeneration.

Overall, the genetic risk variants explained the 23% of disease susceptibility, whereas their integration with non-genetic factors were shown to account for 29% of AMD susceptibility in the Italian population [5]. Furthermore, it is well-known that positive family history (in terms of affected first relatives) for AMD explains 11% of disease risk [13]. To this purpose, these data were employed to realize a computational tool, namely WARE (Wet AMD Risk Evaluation), able to predict the individual risk profile based on the combination of AMD susceptibility factors. In particular, at this stage, WARE is thought to integrate and process genetic and non-genetic variants with an easy-to-use web page interface, aiming to help clinicians in reporting risk assessment upon patient evaluation.

## 2. Materials and Methods

WARE is an open-source, platform-independent, browser-based interface for AMD risk assessment that takes genetic and non-genetic factors into account.

Concerning genetic risk factors (rs1061170, *CFH*; rs10490924, *ARMS2*; rs2227306, *IL8*; rs5749482, *TIMP3*; rs8135665, *SLC16A8*; rs8017304, *RAD51B*; rs943080, *VEGFA*; rs13081855, *COL8A1*) for AMD, the raw OR (odds ratio) measures were retrieved by Cascella et al., 2017 [5]. Such estimates were utilized for calculating the normalized ORs for each risk variant based on their relative frequency in the general population retrieved by the *1000 Genome* database [14]. In particular, such frequencies were multiplied by the raw ORs in order to calculate the average risk of AMD in the general population. Successively, the obtained values were utilized as references to compute the normalized ORs of the above-mentioned genetic risk variants (Table 1).

Concerning the non-genetic risk factors (smoking habits, positive family history for AMD) utilized for the tool development, their related risk estimates were retrieved by Cascella et al., 2017 [5]. Finally, the normalized ORs genetic variants and the ORs for non-genetic risk factors have been integrated in the WARE tool in order to provide the overall AMD risk estimate.

In particular, the tool is written in R programming language (version 4.1.2), and the published interactive app is a dashboard made with Shiny (version 1.6.0). The application can be run locally or on a server. Running WARE requires installing the latest version of R and Rstudio [15]. WARE is freely available on GitHub (https://github.com/Andreater/WARE-AMD-Tool, accessed on 31 May 2022), and it is released under the AGPLv3 license. Accessing the GitHub page, users can download the software and the instruction files, which are reported on the readme file of the repository. Starting and using WARE is particularly easy: after downloading all the program files, the user can open “dashboard.r” using Rstudio and then click on the “Run App” button at the top-right corner of the source pane. Selecting a genotype for each risk factor available in the genetic risk tab, WARE builds a table with the associated OR for that risk factor. When clicking on the “Execute” button, the software calculates the overall risk as the product of all the associated ORs, then updates all plots and text boxes.

## 3. Results and Discussion

**Figure 1:**
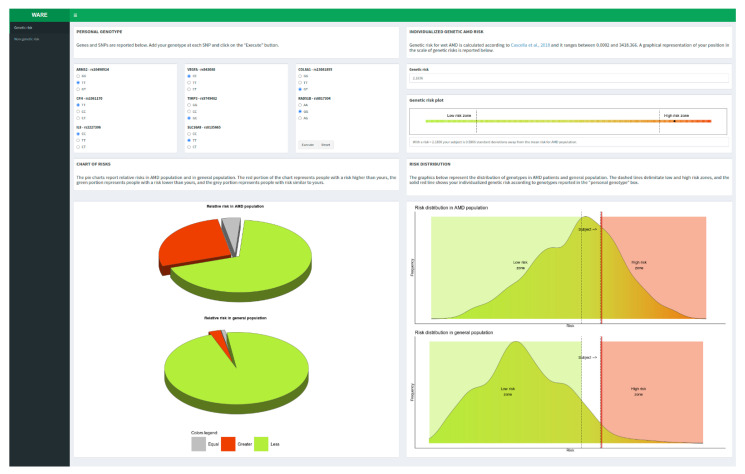
Genetic risk tab [16].

The majority of multifactorial diseases are characterized by the interaction of a large number of genetic and non-genetic factors that typically present ORs in the range of 1.1 to 2.0. However, these ORs are calculated utilizing the lowest-risk allele as a reference (i.e., OR = 1) without considering that all of the possible genotypic classes of genetic variants (homozygous wild-type, homozygous variant, and heterozygous) are normally distributed with their own frequencies within the general population [16]. Indeed, the configuration of a reliable and clinically useful risk profile should combine genetic factors adjusted for the real risk in the general population with the non-genetic risk factors. Taking into consideration the multifactorial features characterizing AMD, the availability of a tool able to provide the individual risk profile is extremely helpful for personalizing the follow-up and treatment protocols of patients. To this purpose, WARE represents an informatic computational tool created to evaluate the individual risk profile of subjects taking into account their genetic architecture, family history, and smoking habit. WARE is a user-friendly software aiming to help clinicians in the evaluation of patients at risk of developing exudative AMD. To this extent, WARE has been designed in a pleasant and easy-to-use web page. The tool is characterized by interfaces that lead users to setting genotypes and non-genetic factors to calculate the risk of wet AMD. In fact, WARE has two dashboard tabs: one for the assessment of genetic risk factors and the other for the non-genetic risk factors. In particular, the genetic risk tab contains five boxes (SNPs, genetic risk, genetic risk plot, chart of risks, and risk distribution) with short descriptions thought to facilitate the usage of the tool and the interpretation of results (Figure 1).

**Figure 2:**
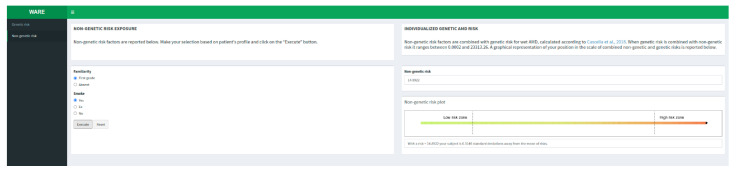
Non-genetic risk tab [16].

Genetic AMD risk is reported as absolute and relative risk with respect to AMD cases and Italian general population. After calculating genetic risk, the user can take into account the non-genetic features (familiarity and smoking habit) in order to predict the overall risk of disease combining genetic and non-genetic factors (Figure 2).

The user can freely move on the two main tabs (namely “Genetic risk” and “Non-genetic risk”) although there are some constraints. In fact, the user cannot obtain a risk estimate if he does not insert a value for all the genotypes in the grid on the genetic risk tab. Moreover, the user cannot obtain the risk estimate on the non-genetic risk tab if he does not insert a value for both the factors in the grid and if he did not calculate the genetic risk first. If the user only knows about one of the two non-genetic risk factors available, and he would like to assess the influence of just one, he can set the lowest level (Familiarity: “Absent”; Smoke: “No”) of the unknown factor before executing the calculation. In fact, the lowest level of both non-genetic factors does not change the overall genetic risk calculated before.

**Figure 3:**
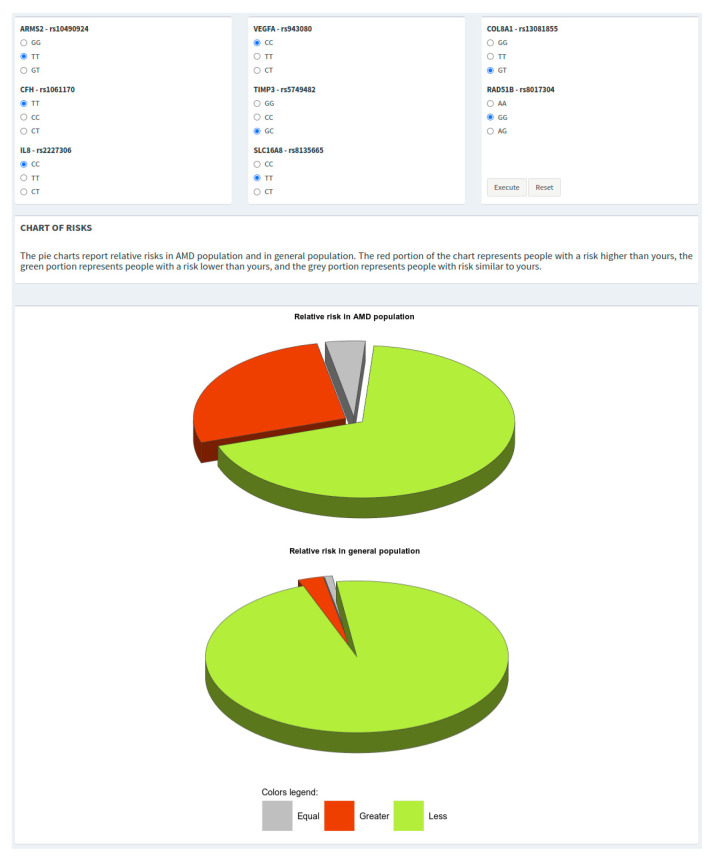
Genotypes grid and pie charts showing the risk of the individual with respect to both AMD and general populations.

All of the plots shown in WARE can be easily copied or saved for reporting purposes just by right-clicking on the figure and selecting the wanted option. In addition, the tool provides two pie charts showing the relative risk with respect to AMD and the general population. The grey slice of the pie shows the portion of subjects with a risk (±0.15) equal to that calculated by the user, while the red and green slices show the portions of subjects with greater or lower risk, respectively (Figure 3).

**Figure 4:**
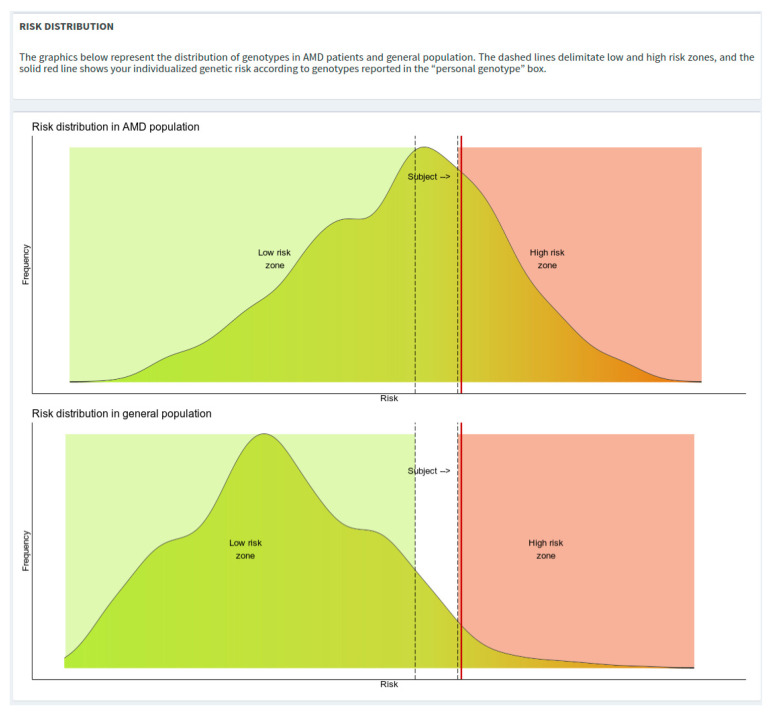
Risk distribution plots with low- and high-risk zones highlighted.

The risk distribution plots depict density curves of the risks from AMD and general population. To avoid potential over/under-estimation of the genetic risk estimate, specific boundaries have been defined as “low-risk zone” (i.e., OR = 0.7) and a “high-risk zone” (i.e., OR = 2.0), represented in the risk distribution plots in green and red colors, respectively (Figure 4).

After the execution of the calculation, a red vertical line will be plotted showing the risk of the subject of interest.

In recent years, a number of tests have been proposed to calculate the risk of developing AMD taking into consideration different variables that affect the predictive variability [17,18,19,20,21,22]. In particular, these predictive tests are mainly composed of a variable number of risk genetic factors, which are generally retrieved from large-scale studies, which often include different populations and AMD phenotypes and do not consider the existence of population-specific genetic, demographic, and lifestyle features, which affect the prevalence of disease as well as the susceptibility to develop the disease. In this regard, the WARE tool was realized taking into account the eight genetic variants (rs1061170, rs10490924, rs2227306, rs5749482, rs8135665, rs8017304, rs943080, rs13081855) specifically associated with wet AMD, which have been validated in the Italian population [5]. In fact, the tool does not include genetic variants (such as polymorphisms of *C2*, *C3*, and *CFB* genes) known to be broadly associated in other populations but not in the Italian cohort. The same rational was used for the selection of non-genetic factors (namely, smoking habit and family history) to be included in the tool, with the aim of providing a comprehensive individual risk profile. However, the tool presents some limitations that deal with the fact that, at the moment, it is able to predict the risk for wet AMD only, and it does not include unknown genetic/non-genetic factors that may likewise contribute to determine the susceptibility to the disease.

To our knowledge, WARE represents a reliable population-specific computational system able to estimate the risk for wet AMD development. It is important to remark that this model can be suitable to compute the disease risk adjusted to different populations considering their specific genetic factors and related frequencies, non-genetic factors, and the disease prevalence.

The tool can rely on a computational system that has been thought to reduce the gap between genetic data and clinical practice. Indeed, it represents a user-friendly and rapid means to provide an individual risk profile for wet AMD, which can be exploited to promote preventive actions and personalized programs for affected patients or at-risk individuals. In fact, WARE allows the assessment of the genetic load associated with wet AMD risk at the individual level and compare it with the average risk observed in the other patients as well as in the general population. In this way, clinicians are able to evaluate if the patient displays particular genetic features that may suggest the need of provide the patient with a personalized approach based on his or her genetic make-up and non-genetic factors.

In this case, the preventive actions are intended as tailored interventions on modifiable risk factors (smoking habits, lifestyle, diet) on the basis of the individual risk zone. Moreover, the stratification of patients by WARE application will allow them to benefit from specific ophthalmological monitoring and personalized follow-up programs. As a result, WARE is expected to improve the management of AMD, promote collaboration among clinical and genetic specialists, and enhance AMD-related services and care provision. In this context, the results of WARE tool should be discussed by a combined counselling provided by both the ophthalmologist and the geneticist. In fact, they should inform patients and family members that the genetic test will not provide a diagnostic response as for many mendelian disorders; rather, it will give an estimation of the individual risk of AMD onset. In this perspective, the test is intended to discriminate between “high-risk” and “low-risk” subjects through a combination of genetic profiles adjusted for the real risk of AMD within the reference population, non-genetic risk factors, and prevalence of the disease. The counselling should therefore be able to provide the patients with a full understanding of the meaning of their individual risk as well as to explain to them which preventive actions and strategies are available for improving the patient’s quality of life. Finally, it is important to remind that the international guidelines about genetic test administration suggest evaluating the clinical usefulness of the test in relation to the single case in order to protect the patient’s needs and avoid harmful behaviors.

In the era of personalized medicine, the WARE tool represents a proactive tool that could be integrated with ophthalmological data and additional genetic/non-genetic data to provide an additional support for the decision-making process of both clinicians and patients. In addition, WARE could be integrated with pharmacogenetic data in order to predict not only the AMD risk but also the individual response to anti-VEGF treatments and the risk of relapse in patients treated with anti-VEGF.

## Figures and Tables

**Table 1:** Genetic risk variants and their normalized ORs employed by WARE.

Gene	SNPs	ORs
*ARMS2*	rs10490924 G/T	GG = 0.12 (0.08–0.17)
		TT = 8.3 (5.73–12.04)
		GT = 2.38 (1.95–2.90)
*CFH*	rs1061170 T/C	TT = 0.19 (0.14–0.25)
		CC = 5.16 (3.90–6.82)
		CT = 2.19 (1.76–2.72)
*IL8*	rs2227306 C/T	CC = 0.53 (0.39–0.72)
		TT = 1.87 (1.38–2.53)
		CT = 1.32 (1.08–1.59)
*VEGFA*	rs943080 C/T	CC = 0.59 (0.45–0.77)
		TT = 1.69 (1.29–2.19)
		CT = 1.23 (0.97–1.55)
*TIMP3*	rs5749482 C/G	GG = 1.56 (0.77–3.16)
		CC = 0.63 (0.31–1.29)
		GC = 1.00 (0.45–1.91)
*SLC16A8*	rs8135665 C/T	CC = 0.44 (0.28–0.69)
		TT = 2.25 (1.44–3.51)
		CT = 1.59 (1.30–1.95)
*COL8A1*	rs13081855 G/T	GG = 0.16 (0.04–0.55)
		TT = 6.19 (1.78–21.49)
		GT = 1.85 (1.43–2.39)
*RAD51B*	rs8017304 A/G	AA = 0.69 (0.52–0.91)
		GG = 1.44 (1.10–1.88)
		AG = 1.15 (0.94–1.40)
